# 1,5-Dimethyl-4-{[1-(3-methyl-5-oxo-1-phenyl-4,5-dihydro-1*H*-pyrazol-4-yl­idene)eth­yl]amino}-2-phenyl-1*H*-pyrazol-3(2*H*)-one

**DOI:** 10.1107/S1600536810020532

**Published:** 2010-06-05

**Authors:** Hualing Zhu, Jun Shi, Zhen Wei, Yanan Bai, Luxia Bu

**Affiliations:** a Department of Basic Science, Tianjin Agricultural College, Tianjin Jinjing Road No. 22, Tianjin 300384, People’s Republic of China

## Abstract

In the title compound, C_23_H_23_N_5_O_2_, an intra­molecular N—H⋯O hydrogen bond generates an *S*(6) ring, and the dihedral angle between the pyrazole rings is 48.42 (8)°. The dihedral angles between the pyrazole rings and their attached phenyl rings are 10.06 (8) and 47.53 (8)°.

## Related literature

For related structures and background references, see: Zhang *et al.* (2010 ▶); Zhu *et al.* (2010 ▶).
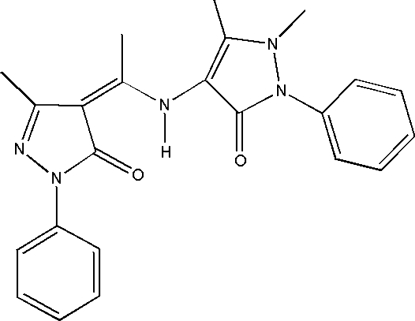


## Experimental

### 

#### Crystal data

C_23_H_23_N_5_O_2_
*M* *_r_* = 401.46
Monoclinic, 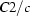

*a* = 20.486 (4) Å
*b* = 10.209 (2) Å
*c* = 19.753 (4) Å
β = 102.76 (3)°
*V* = 4029.3 (14) Å^3^
*Z* = 8
Mo *K*α radiation
μ = 0.09 mm^−1^
*T* = 113 K
0.20 × 0.18 × 0.12 mm

#### Data collection

Rigaku Saturn CCD diffractometer
Absorption correction: multi-scan (*CrystalClear*; Rigaku, 2005 ▶) *T*_min_ = 0.983, *T*_max_ = 0.990
13299 measured reflections
3553 independent reflections
2858 reflections with *I* > 2σ(*I*)
*R*_int_ = 0.038

#### Refinement

*R*[*F* ^2^ > 2σ(*F* ^2^)] = 0.040
*wR*(*F* ^2^) = 0.105
*S* = 1.00
3553 reflections
280 parameters
1 restraint
H atoms treated by a mixture of independent and constrained refinement
Δρ_max_ = 0.24 e Å^−3^
Δρ_min_ = −0.20 e Å^−3^

### 

Data collection: *CrystalClear* (Rigaku, 2005 ▶); cell refinement: *CrystalClear*; data reduction: *CrystalClear*; program(s) used to solve structure: *SHELXS97* (Sheldrick, 2008 ▶); program(s) used to refine structure: *SHELXL97* (Sheldrick, 2008 ▶); molecular graphics: *SHELXTL* (Sheldrick, 2008 ▶); software used to prepare material for publication: *SHELXTL*.

## Supplementary Material

Crystal structure: contains datablocks I, global. DOI: 10.1107/S1600536810020532/hb5465sup1.cif

Structure factors: contains datablocks I. DOI: 10.1107/S1600536810020532/hb5465Isup2.hkl

Additional supplementary materials:  crystallographic information; 3D view; checkCIF report

## Figures and Tables

**Table 1:** Hydrogen-bond geometry (Å, °)

*D*—H⋯*A*	*D*—H	H⋯*A*	*D*⋯*A*	*D*—H⋯*A*
N3—H3*A*⋯O1	0.90 (1)	1.85 (1)	2.6459 (15)	146 (2)

**Fig. 1.**
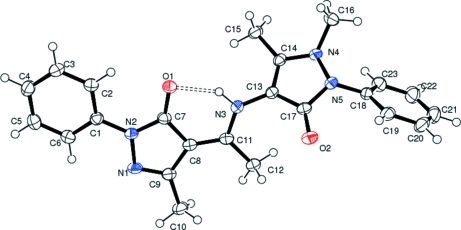
The molecular structure of (I) with displacement ellipsoids drawn at the 30% probability level. H atoms are presented as spheres of arbitrary radius.

## supplementary crystallographic information

### Comment

As part of our onging studies of pyrazole derivatives (Zhu *et al.*,  2010), we now report the structure of the title compound, (I).

In the molecule of the title compound, (Fig.1) there is one molecule in the asymmetric unit. Atom O1, C7, C8, C11 and atom N3 form a plane, the largest deviation being 0.0132 (15) Å for atom C11. The dihedral angle between this plane and the pyrazolone ring of PMAP is 0.46 (4)°, indicating that they are essentially coplanar, as seen in Ethyl 2–{[(1*Z*)–(3– methyl–5–oxo–1–phenyl–4,5–dihydro–1*H*–pyrazol–4–ylidene) (*p*–tolyl)methyl]amino}–3–phenylpropanoate (1.52 (4)°; Zhang *et al.*, 2010). The bond lengths within this part of the molecular lie between classical single–and double–bond lengths, indicating extensive conjugation. Atoms N5,N4, C14, C13,C17 and O2 are also nearly coplanar, the largest deviation being 0.0417 (15) Å for atom C14.The dihedral angle between this plane and the phenyl ring of antipyrine is 47.41 (5)°, A strong intramolecular hydrogen bond N3—H3···O1 is observed (Table 1 & Fig. 1), stabilizing to an enamine–keto form.

### Experimental

A mixture of HPMAP (15*m* mol) and 4-antipyrine (15*m* mol) in ethanol (100 ml) was refluxed over a steam bath for about 4 h, then the solution was cooled down to room temperature. After one day, pale yellow blocks were obtained and dried in air. The product was recrystallized from ethanol which afforded pale yellow blocks of (I).

### Refinement

The N-bound H atom was located in a difference map and freely refined. All C-bound H atoms were geometrically positioned and refined using a riding model, with C—H = 0.95 Å for the aryl, 0.98 Å for the methyl H atoms. *U*_iso_(H)= 1.2 *U*_eq_(C) for aryl, 1.5*U*_eq_(C) for methyl H atoms.

### Crystal data

**Table:**

C_23_H_23_N_5_O_2_	*F*(000) = 1696
*M**_r_* = 401.46	*D*_x_ = 1.324 Mg m^−^^3^
Monoclinic, *C*2/*c*	Mo *K*α radiation, λ = 0.71073 Å
Hall symbol: -C 2yc	Cell parameters from 5835 reflections
*a* = 20.486 (4) Å	θ = 2.0–27.9°
*b* = 10.209 (2) Å	µ = 0.09 mm^−^^1^
*c* = 19.753 (4) Å	*T* = 113 K
β = 102.76 (3)°	Block, pale yellow
*V* = 4029.3 (14) Å^3^	0.20 × 0.18 × 0.12 mm
*Z* = 8	

### Data collection

**Table:**

Rigaku Saturn CCD diffractometer	3553 independent reflections
Radiation source: rotating anode	2858 reflections with *I* > 2σ(*I*)
confocal	*R*_int_ = 0.038
Detector resolution: 7.31 pixels mm^-1^	θ_max_ = 25.0°, θ_min_ = 2.0°
ω and φ scans	*h* = −24→21
Absorption correction: multi-scan (*CrystalClear*; Rigaku, 2005)	*k* = −11→12
*T*_min_ = 0.983, *T*_max_ = 0.990	*l* = −23→20
13299 measured reflections	

### Refinement

**Table:**

Refinement on *F*^2^	Secondary atom site location: difference Fourier map
Least-squares matrix: full	Hydrogen site location: inferred from neighbouring sites
*R*[*F*^2^ > 2σ(*F*^2^)] = 0.040	H atoms treated by a mixture of independent and constrained refinement
*wR*(*F*^2^) = 0.105	*w* = 1/[σ^2^(*F*_o_^2^) + (0.0698*P*)^2^] where *P* = (*F*_o_^2^ + 2*F*_c_^2^)/3
*S* = 1.00	(Δ/σ)_max_ = 0.001
3553 reflections	Δρ_max_ = 0.24 e Å^−^^3^
280 parameters	Δρ_min_ = −0.20 e Å^−^^3^
1 restraint	Extinction correction: *SHELXL97* (Sheldrick, 2008), Fc^*^=kFc[1+0.001xFc^2^λ^3^/sin(2θ)]^-1/4^
Primary atom site location: structure-invariant direct methods	Extinction coefficient: 0.0113 (7)

### Special details

**Table:**

Geometry. All e.s.d.'s (except the e.s.d. in the dihedral angle between two l.s. planes) are estimated using the full covariance matrix. The cell e.s.d.'s are taken into account individually in the estimation of e.s.d.'s in distances, angles and torsion angles; correlations between e.s.d.'s in cell parameters are only used when they are defined by crystal symmetry. An approximate (isotropic) treatment of cell e.s.d.'s is used for estimating e.s.d.'s involving l.s. planes.
Refinement. Refinement of *F*^2^ against ALL reflections. The weighted *R*-factor *wR* and goodness of fit *S* are based on *F*^2^, conventional *R*-factors *R* are based on *F*, with *F* set to zero for negative *F*^2^. The threshold expression of *F*^2^ > σ(*F*^2^) is used only for calculating *R*-factors(gt) *etc*. and is not relevant to the choice of reflections for refinement. *R*-factors based on *F*^2^ are statistically about twice as large as those based on *F*, and *R*- factors based on ALL data will be even larger.

### Fractional atomic coordinates and isotropic or equivalent isotropic displacement parameters (Å^2^)

**Table:**

	*x*	*y*	*z*	*U*_iso_*/*U*_eq_	
O1	0.36779 (5)	0.46740 (9)	−0.06459 (5)	0.0331 (3)	
O2	0.30384 (5)	0.76857 (9)	0.14645 (5)	0.0320 (3)	
N1	0.48756 (6)	0.27136 (11)	0.04420 (6)	0.0290 (3)	
N2	0.44223 (6)	0.29879 (11)	−0.01884 (6)	0.0258 (3)	
N3	0.36650 (6)	0.64889 (11)	0.03072 (6)	0.0275 (3)	
N4	0.31259 (6)	0.98296 (10)	0.01180 (6)	0.0265 (3)	
N5	0.29411 (6)	0.95272 (11)	0.07449 (6)	0.0267 (3)	
C1	0.44049 (7)	0.21464 (12)	−0.07603 (7)	0.0250 (3)	
C2	0.39265 (7)	0.23181 (13)	−0.13788 (7)	0.0302 (4)	
H2	0.3599	0.2988	−0.1414	0.036*	
C3	0.39318 (8)	0.15100 (13)	−0.19403 (8)	0.0340 (4)	
H3	0.3608	0.1633	−0.2361	0.041*	
C4	0.44038 (8)	0.05234 (14)	−0.18963 (8)	0.0334 (4)	
H4	0.4408	−0.0021	−0.2285	0.040*	
C5	0.48688 (8)	0.03395 (13)	−0.12788 (8)	0.0325 (4)	
H5	0.5188	−0.0346	−0.1243	0.039*	
C6	0.48738 (7)	0.11448 (13)	−0.07107 (8)	0.0284 (3)	
H6	0.5196	0.1012	−0.0290	0.034*	
C7	0.40969 (7)	0.41613 (13)	−0.01570 (7)	0.0252 (3)	
C8	0.43465 (7)	0.46416 (12)	0.05373 (7)	0.0246 (3)	
C9	0.48298 (7)	0.36845 (13)	0.08621 (7)	0.0265 (3)	
C10	0.52713 (8)	0.36606 (15)	0.15746 (8)	0.0383 (4)	
H10A	0.5499	0.2812	0.1655	0.057*	
H10B	0.4999	0.3793	0.1919	0.057*	
H10C	0.5605	0.4361	0.1617	0.057*	
C11	0.41305 (7)	0.58307 (13)	0.07650 (7)	0.0242 (3)	
C12	0.43823 (8)	0.63774 (13)	0.14760 (7)	0.0296 (4)	
H12A	0.4385	0.7336	0.1453	0.044*	
H12B	0.4838	0.6061	0.1663	0.044*	
H12C	0.4089	0.6094	0.1779	0.044*	
C13	0.34091 (7)	0.77511 (12)	0.03765 (7)	0.0236 (3)	
C14	0.33636 (7)	0.87041 (12)	−0.01143 (7)	0.0238 (3)	
C15	0.35030 (8)	0.86237 (14)	−0.08178 (7)	0.0302 (4)	
H15A	0.3841	0.7946	−0.0824	0.045*	
H15B	0.3670	0.9471	−0.0940	0.045*	
H15C	0.3090	0.8401	−0.1155	0.045*	
C16	0.26801 (8)	1.07262 (14)	−0.03498 (8)	0.0340 (4)	
H16A	0.2931	1.1167	−0.0653	0.051*	
H16B	0.2502	1.1381	−0.0076	0.051*	
H16C	0.2309	1.0229	−0.0634	0.051*	
C17	0.31227 (7)	0.82187 (13)	0.09295 (7)	0.0252 (3)	
C18	0.29907 (7)	1.05520 (13)	0.12492 (7)	0.0268 (3)	
C19	0.26278 (7)	1.04378 (15)	0.17587 (7)	0.0325 (4)	
H19	0.2345	0.9703	0.1765	0.039*	
C20	0.26814 (8)	1.14062 (16)	0.22600 (8)	0.0412 (4)	
H20	0.2439	1.1326	0.2616	0.049*	
C21	0.30837 (9)	1.24844 (17)	0.22456 (9)	0.0454 (5)	
H21	0.3116	1.3147	0.2589	0.054*	
C22	0.34389 (9)	1.25982 (15)	0.17310 (9)	0.0429 (4)	
H22	0.3712	1.3345	0.1718	0.052*	
C23	0.33989 (8)	1.16264 (13)	0.12312 (8)	0.0344 (4)	
H23	0.3649	1.1698	0.0881	0.041*	
H3A	0.3562 (8)	0.6116 (14)	−0.0115 (6)	0.045 (5)*	

### Atomic displacement parameters (Å^2^)

**Table:**

	*U*^11^	*U*^22^	*U*^33^	*U*^12^	*U*^13^	*U*^23^
O1	0.0351 (6)	0.0329 (5)	0.0257 (6)	0.0095 (5)	−0.0053 (5)	−0.0022 (4)
O2	0.0367 (6)	0.0363 (6)	0.0236 (6)	−0.0021 (5)	0.0081 (5)	0.0049 (5)
N1	0.0286 (7)	0.0338 (7)	0.0217 (7)	0.0052 (5)	−0.0003 (5)	0.0042 (5)
N2	0.0265 (7)	0.0284 (6)	0.0198 (6)	0.0039 (5)	−0.0006 (5)	0.0006 (5)
N3	0.0322 (7)	0.0267 (6)	0.0216 (6)	0.0026 (5)	0.0016 (6)	−0.0033 (5)
N4	0.0322 (7)	0.0294 (6)	0.0186 (6)	0.0050 (5)	0.0073 (5)	0.0030 (5)
N5	0.0322 (7)	0.0296 (6)	0.0198 (6)	0.0045 (5)	0.0092 (5)	0.0016 (5)
C1	0.0270 (8)	0.0253 (7)	0.0230 (8)	−0.0017 (6)	0.0063 (6)	0.0005 (6)
C2	0.0311 (9)	0.0308 (7)	0.0264 (8)	0.0043 (6)	0.0011 (7)	0.0012 (6)
C3	0.0401 (9)	0.0357 (8)	0.0236 (8)	0.0003 (7)	0.0012 (7)	−0.0004 (6)
C4	0.0404 (10)	0.0324 (8)	0.0296 (9)	−0.0027 (7)	0.0126 (7)	−0.0045 (7)
C5	0.0318 (9)	0.0279 (7)	0.0398 (9)	0.0030 (6)	0.0123 (7)	−0.0008 (7)
C6	0.0271 (8)	0.0280 (7)	0.0291 (8)	0.0001 (6)	0.0038 (6)	0.0032 (6)
C7	0.0234 (8)	0.0276 (7)	0.0232 (8)	0.0020 (6)	0.0019 (6)	0.0021 (6)
C8	0.0240 (8)	0.0281 (7)	0.0207 (8)	−0.0008 (6)	0.0027 (6)	0.0025 (6)
C9	0.0261 (8)	0.0310 (7)	0.0223 (7)	0.0017 (6)	0.0049 (6)	0.0035 (6)
C10	0.0404 (10)	0.0485 (9)	0.0226 (8)	0.0144 (8)	−0.0001 (7)	0.0007 (7)
C11	0.0236 (8)	0.0271 (7)	0.0213 (7)	−0.0031 (6)	0.0035 (6)	0.0035 (6)
C12	0.0313 (8)	0.0320 (8)	0.0233 (8)	0.0014 (6)	0.0015 (6)	−0.0008 (6)
C13	0.0239 (7)	0.0248 (7)	0.0209 (7)	−0.0005 (6)	0.0023 (6)	−0.0013 (6)
C14	0.0207 (7)	0.0298 (7)	0.0200 (7)	0.0001 (6)	0.0025 (6)	−0.0027 (6)
C15	0.0316 (9)	0.0378 (8)	0.0213 (8)	0.0026 (7)	0.0061 (6)	0.0007 (6)
C16	0.0387 (9)	0.0323 (8)	0.0308 (9)	0.0100 (7)	0.0073 (7)	0.0076 (7)
C17	0.0237 (8)	0.0294 (7)	0.0207 (7)	−0.0022 (6)	0.0009 (6)	0.0006 (6)
C18	0.0266 (8)	0.0315 (7)	0.0205 (7)	0.0087 (6)	0.0015 (6)	−0.0018 (6)
C19	0.0276 (8)	0.0443 (9)	0.0244 (8)	0.0095 (7)	0.0030 (6)	−0.0002 (7)
C20	0.0355 (10)	0.0633 (11)	0.0232 (8)	0.0165 (8)	0.0029 (7)	−0.0056 (8)
C21	0.0475 (11)	0.0484 (10)	0.0339 (10)	0.0148 (8)	−0.0048 (8)	−0.0161 (8)
C22	0.0455 (11)	0.0378 (9)	0.0407 (10)	0.0037 (8)	−0.0009 (8)	−0.0095 (8)
C23	0.0340 (9)	0.0354 (8)	0.0328 (9)	0.0044 (7)	0.0052 (7)	−0.0015 (7)

### Geometric parameters (Å, °)

**Table:**

O1—C7	1.2559 (17)	C9—C10	1.496 (2)
O2—C17	1.2341 (16)	C10—H10A	0.9800
N1—C9	1.3093 (18)	C10—H10B	0.9800
N1—N2	1.4073 (16)	C10—H10C	0.9800
N2—C7	1.3792 (17)	C11—C12	1.4933 (19)
N2—C1	1.4133 (17)	C12—H12A	0.9800
N3—C11	1.3408 (18)	C12—H12B	0.9800
N3—C13	1.4091 (17)	C12—H12C	0.9800
N3—H3A	0.899 (9)	C13—C14	1.3622 (19)
N4—C14	1.3664 (16)	C13—C17	1.4314 (19)
N4—N5	1.4068 (16)	C14—C15	1.4814 (19)
N4—C16	1.4673 (18)	C15—H15A	0.9800
N5—C17	1.4128 (17)	C15—H15B	0.9800
N5—C18	1.4327 (17)	C15—H15C	0.9800
C1—C6	1.391 (2)	C16—H16A	0.9800
C1—C2	1.398 (2)	C16—H16B	0.9800
C2—C3	1.384 (2)	C16—H16C	0.9800
C2—H2	0.9500	C18—C19	1.382 (2)
C3—C4	1.386 (2)	C18—C23	1.385 (2)
C3—H3	0.9500	C19—C20	1.386 (2)
C4—C5	1.385 (2)	C19—H19	0.9500
C4—H4	0.9500	C20—C21	1.379 (3)
C5—C6	1.3893 (19)	C20—H20	0.9500
C5—H5	0.9500	C21—C22	1.379 (3)
C6—H6	0.9500	C21—H21	0.9500
C7—C8	1.4396 (19)	C22—C23	1.389 (2)
C8—C11	1.4000 (19)	C22—H22	0.9500
C8—C9	1.4373 (19)	C23—H23	0.9500
			
C9—N1—N2	106.53 (11)	N3—C11—C12	119.56 (12)
C7—N2—N1	111.35 (10)	C8—C11—C12	123.93 (12)
C7—N2—C1	129.57 (11)	C11—C12—H12A	109.5
N1—N2—C1	118.78 (11)	C11—C12—H12B	109.5
C11—N3—C13	128.11 (12)	H12A—C12—H12B	109.5
C11—N3—H3A	113.6 (11)	C11—C12—H12C	109.5
C13—N3—H3A	117.5 (11)	H12A—C12—H12C	109.5
C14—N4—N5	107.23 (10)	H12B—C12—H12C	109.5
C14—N4—C16	122.11 (12)	C14—C13—N3	123.54 (13)
N5—N4—C16	116.12 (11)	C14—C13—C17	109.25 (12)
N4—N5—C17	109.15 (10)	N3—C13—C17	127.10 (12)
N4—N5—C18	117.40 (11)	C13—C14—N4	109.58 (12)
C17—N5—C18	122.47 (11)	C13—C14—C15	129.12 (12)
C6—C1—C2	119.56 (13)	N4—C14—C15	121.23 (12)
C6—C1—N2	119.69 (13)	C14—C15—H15A	109.5
C2—C1—N2	120.74 (12)	C14—C15—H15B	109.5
C3—C2—C1	119.82 (13)	H15A—C15—H15B	109.5
C3—C2—H2	120.1	C14—C15—H15C	109.5
C1—C2—H2	120.1	H15A—C15—H15C	109.5
C2—C3—C4	120.79 (14)	H15B—C15—H15C	109.5
C2—C3—H3	119.6	N4—C16—H16A	109.5
C4—C3—H3	119.6	N4—C16—H16B	109.5
C5—C4—C3	119.24 (14)	H16A—C16—H16B	109.5
C5—C4—H4	120.4	N4—C16—H16C	109.5
C3—C4—H4	120.4	H16A—C16—H16C	109.5
C4—C5—C6	120.81 (13)	H16B—C16—H16C	109.5
C4—C5—H5	119.6	O2—C17—N5	124.01 (13)
C6—C5—H5	119.6	O2—C17—C13	131.67 (13)
C5—C6—C1	119.75 (14)	N5—C17—C13	104.32 (11)
C5—C6—H6	120.1	C19—C18—C23	120.71 (13)
C1—C6—H6	120.1	C19—C18—N5	118.54 (13)
O1—C7—N2	125.62 (12)	C23—C18—N5	120.74 (13)
O1—C7—C8	128.99 (13)	C18—C19—C20	119.31 (15)
N2—C7—C8	105.38 (11)	C18—C19—H19	120.3
C11—C8—C9	132.97 (13)	C20—C19—H19	120.3
C11—C8—C7	122.03 (12)	C21—C20—C19	120.52 (16)
C9—C8—C7	104.97 (12)	C21—C20—H20	119.7
N1—C9—C8	111.75 (12)	C19—C20—H20	119.7
N1—C9—C10	118.09 (12)	C20—C21—C22	119.82 (15)
C8—C9—C10	130.15 (13)	C20—C21—H21	120.1
C9—C10—H10A	109.5	C22—C21—H21	120.1
C9—C10—H10B	109.5	C21—C22—C23	120.38 (16)
H10A—C10—H10B	109.5	C21—C22—H22	119.8
C9—C10—H10C	109.5	C23—C22—H22	119.8
H10A—C10—H10C	109.5	C18—C23—C22	119.25 (15)
H10B—C10—H10C	109.5	C18—C23—H23	120.4
N3—C11—C8	116.50 (12)	C22—C23—H23	120.4
			
C9—N1—N2—C7	0.73 (15)	C9—C8—C11—N3	179.24 (14)
C9—N1—N2—C1	175.01 (12)	C7—C8—C11—N3	1.6 (2)
C14—N4—N5—C17	5.07 (15)	C9—C8—C11—C12	−1.4 (2)
C16—N4—N5—C17	145.59 (12)	C7—C8—C11—C12	−179.06 (13)
C14—N4—N5—C18	150.16 (12)	C11—N3—C13—C14	130.25 (16)
C16—N4—N5—C18	−69.32 (15)	C11—N3—C13—C17	−53.9 (2)
C7—N2—C1—C6	167.10 (13)	N3—C13—C14—N4	−177.17 (12)
N1—N2—C1—C6	−5.98 (19)	C17—C13—C14—N4	6.37 (16)
C7—N2—C1—C2	−12.1 (2)	N3—C13—C14—C15	5.8 (2)
N1—N2—C1—C2	174.78 (12)	C17—C13—C14—C15	−170.70 (13)
C6—C1—C2—C3	−1.4 (2)	N5—N4—C14—C13	−7.03 (15)
N2—C1—C2—C3	177.88 (13)	C16—N4—C14—C13	−144.66 (13)
C1—C2—C3—C4	0.4 (2)	N5—N4—C14—C15	170.30 (12)
C2—C3—C4—C5	0.8 (2)	C16—N4—C14—C15	32.7 (2)
C3—C4—C5—C6	−1.1 (2)	N4—N5—C17—O2	178.14 (12)
C4—C5—C6—C1	0.2 (2)	C18—N5—C17—O2	35.2 (2)
C2—C1—C6—C5	1.0 (2)	N4—N5—C17—C13	−1.27 (14)
N2—C1—C6—C5	−178.23 (12)	C18—N5—C17—C13	−144.24 (13)
N1—N2—C7—O1	178.08 (13)	C14—C13—C17—O2	177.62 (14)
C1—N2—C7—O1	4.6 (2)	N3—C13—C17—O2	1.3 (2)
N1—N2—C7—C8	−1.39 (15)	C14—C13—C17—N5	−3.03 (15)
C1—N2—C7—C8	−174.88 (13)	N3—C13—C17—N5	−179.33 (13)
O1—C7—C8—C11	0.3 (2)	N4—N5—C18—C19	159.88 (12)
N2—C7—C8—C11	179.69 (12)	C17—N5—C18—C19	−59.97 (18)
O1—C7—C8—C9	−177.98 (14)	N4—N5—C18—C23	−20.81 (19)
N2—C7—C8—C9	1.45 (15)	C17—N5—C18—C23	119.34 (15)
N2—N1—C9—C8	0.27 (16)	C23—C18—C19—C20	−0.7 (2)
N2—N1—C9—C10	−178.89 (13)	N5—C18—C19—C20	178.58 (13)
C11—C8—C9—N1	−179.06 (14)	C18—C19—C20—C21	1.1 (2)
C7—C8—C9—N1	−1.10 (16)	C19—C20—C21—C22	−0.4 (2)
C11—C8—C9—C10	0.0 (3)	C20—C21—C22—C23	−0.7 (2)
C7—C8—C9—C10	177.93 (15)	C19—C18—C23—C22	−0.3 (2)
C13—N3—C11—C8	−173.48 (13)	N5—C18—C23—C22	−179.56 (13)
C13—N3—C11—C12	7.1 (2)	C21—C22—C23—C18	1.0 (2)

### Hydrogen-bond geometry (Å, °)

**Table:**

*D*—H···*A*	*D*—H	H···*A*	*D*···*A*	*D*—H···*A*
N3—H3A···O1	0.90 (1)	1.85 (1)	2.6459 (15)	146 (2)
